# Tracing ALS Degeneration: Insights from Spinal Cord and Cortex Transcriptomes

**DOI:** 10.3390/genes15111431

**Published:** 2024-11-02

**Authors:** Nela Pragathi Sneha, S. Akila Parvathy Dharshini, Y.-h. Taguchi, M. Michael Gromiha

**Affiliations:** 1 Department of Biotechnology, Bhupat and Jyoti Mehta School of Biosciences, Indian Institute of Technology Madras, Chennai 600036, Tamilnadu, India; bt18d019@smail.iitm.ac.in (N.P.S.); akilabioinfo@gmail.com (S.A.P.D.); 2 Department of Physics, Chuo University, Kasuga, Bunkyo-ku, Tokyo 112-8551, Japan; tag@granular.com

**Keywords:** ALS, neurodegenerative diseases, transcriptome analysis, gene expression, network

## Abstract

Background/Objectives: Amyotrophic Lateral Sclerosis is a progressive neurodegenerative disorder characterized by the loss of upper and lower motor neurons. Key factors contributing to neuronal death include mitochondrial energy damage, oxidative stress, and excitotoxicity. The frontal cortex is crucial for action initiation, planning, and voluntary movements whereas the spinal cord facilitates communication with the brain, walking, and reflexes. By investigating transcriptome data from the frontal cortex and spinal cord, we aim to elucidate common pathological mechanisms and pathways involved in ALS for understanding the disease progression and identifying potential therapeutic targets. Methods: In this study, we quantified gene and transcript expression patterns, predicted variants, and assessed their functional effects using computational tools. It also includes predicting variant-associated regulatory effects, constructing functional interaction networks, and performing a gene enrichment analysis. Results: We found novel genes for the upregulation of immune response, and the downregulation of metabolic-related and defective degradation processes in both the spinal cord and frontal cortex. Additionally, we observed the dysregulation of histone regulation and blood pressure-related genes specifically in the frontal cortex. Conclusions: These results highlight the distinct and shared molecular disruptions in ALS, emphasizing the critical roles of immune response and metabolic dysfunction in neuronal degeneration. Targeting these pathways may provide new therapeutic avenues to combat neurodegeneration and preserve neuronal health.

## 1. Introduction

ALS (Amyotrophic Lateral Sclerosis) is a fatal neurodegenerative disorder that affects both the upper and lower motor neurons of the CNS. Symptoms of ALS are broadly classified into four types: respiratory symptoms (restricted breathing, shortness of breath), bulbar symptoms (swallowing, excess saliva), upper body symptoms (handwriting, upper body muscle spasms), and lower body symptoms (climbing stairs, walking). This disease is a progressive neurodegenerative disorder and the patient dies within 3 to 5 years from the time of onset of symptoms. There is no disease-modifying therapy for ALS other than respiratory support and treatments to ease the symptoms. FDA-approved drugs for ALS include Riluzole [1] and Edavarone [2], which provide limited positive outcomes for patients. The major types of ALS are familial ALS and sporadic ALS (sALS). Most of the cases are sporadic and some cases have an inherited background. C9orf72 (chromosome 9 open reading frame 72), TDP-43 (transactive response DNA binding protein 43), SOD1 (copper–zinc superoxide dismutase 1), FUS (fused in sarcoma), UBQLN2 (Ubiquilin-2), OPTN (Optineurin), and TBK1 (tank-binding kinase 1) are some of the well-known genes in which the genetic variants are disease-associated. The regions that are primarily affected in ALS patients are the cortex, brainstem, and spinal cord. Neurons that are projected from the cortex to the brainstem/spinal cord (upper motor neurons) and projecting from the brainstem to muscles (lower motor neurons) are degenerated in ALS. These regions form a well-connected network and neuronal atrophy is observed in the cortico-spinal tract and cortico-bulbar tract. The frontal cortex (FC) and spinal cord (SC) work together in a coordinated manner to control voluntary movements. The frontal cortex initiates and plans movements, and the spinal cord executes these movements by transmitting signals to the muscles through motor neurons. Investigating the transcriptome patterns in the spinal cord and frontal cortex of individuals with Amyotrophic Lateral Sclerosis (ALS) compared to control subjects can provide valuable insights into the molecular mechanisms underlying neurodegeneration. Earlier studies on ALS revealed biomarkers from human- and mouse-based transcriptomic studies [3]. Major genes that were identified are supported by RNA seq studies, making them palliative targets in mechanisms like calcium metabolism, RNA metabolism, and pathways supporting neuroinflammation [4]. Genes such as EATT2 (Excitatory amino acid transporter 2), CHCHD10 (Coiled-Coil-Helix-Coiled-Coil-Helix Domain Containing 10), ANXA11 (Annexin A11), VAPB (Vesicle-Associated Membrane Protein), DCTN1 (Dynactin Subunit 1), OPTN (Optineurin), ADAR2 (Adenosine deaminases acting on RNA 2), and SETX(Senataxin) have been identified as potential candidates for splicing events [5]. There are evident studies on the impact of splicing events on neuron differentiation in ALS [5,6]. Genes associated with oligodendrocytes, astrocytes, and glial cell types have been shown to significantly influence pathways related to energy metabolism and contribute to increased neuronal loss. For example, Provenzano et al. (2023) [7] discussed the role of astrocytes in glutamate excitotoxicity in ALS, highlighting their toxic effects. Oligodendrocytes are implicated in motor neuron death in ALS patients due to their failure to provide adequate metabolic support and myelinate axons. In ALS, microglia have both neuroprotective and inflammatory roles, suggesting their potential inclusion in multi-drug therapies [8]. Previous studies using microarray, RNA-seq, and single-cell sequencing data have provided valuable insights into gene expression patterns, alternative splicing events, and cell-level expression [3].

Previous RNA-seq studies of human spinal cord post mortem samples have shown that upregulated genes are often associated with neuroinflammation and neuron death. Conversely, downregulated genes, such as SNAP25 (Synaptosome Associated Protein 25), SNPH (Syntaphilin), STX1B (syntaxin 1B), and SYT4 (Synaptotagmin 4), are involved in synaptic function [9]. In a study by MacLean et al. (2021) [10], motor neurons in the spinal cords of SOD1(superoxide dismutase 1) mice were analyzed using single-nucleus RNA-seq, revealing that disrupted neuron–glia communication contributes to neuron loss in ALS. This study identified downregulated genes like Epha3 that play a role in neuroprotective mechanisms. Additionally, microarray studies on mouse spinal cords have highlighted dysregulated genes, such as GFAP (Glial fibrillary acidic protein) and ITGAM(Integrin alpha M), which mediate neuronal cell loss and promote microglial proliferation [11]. Recent research has focused on changes in gene expression and the associated functional losses or gains. However, these studies often overlook the impact of transcriptome variants and their potential regulatory effects. To fully understand disease mechanisms, it is essential to analyze differentially expressed genes in conjunction with variant genes (VGs) and their role in regulatory processes. This approach will help interpret the relationships between gene expression changes and genetic variants, uncovering regulatory interactions and their implications for disease.

In this study, we analyzed differentially expressed genes (DEGs) from frontal cortex and spinal cord samples, which are predominantly involved in immune response pathways, identifying them as potential therapeutic targets. We utilized samples from both sporadic ALS (sALS) and C9orf72 ALS (c9ALS) sourced from public datasets. Our investigation focused on differential expression at both gene and transcript levels to elucidate changes in transcriptomic patterns in ALS. We identified variations that affect the regulatory binding of transcription factors, miRNAs, and epigenetic modifications. To explore the functional relevance and disrupted pathways associated with these variants and DEGs, we conducted functional and pathway enrichment analyses. Our findings highlight that the novel genes are chiefly involved in neuroinflammatory responses and the regulation of metabolic and oxidative stress responses. Additionally, some genes play roles in muscle contraction and trans-synaptic signaling, which has been previously linked to motor neuron death in ALS [12]. These genes are also implicated in oxidative stress responses, which are critical in ALS pathology. Antioxidant strategies aimed at managing oxidative stress are being investigated as potential therapies [13]. Neuroinflammation and immune responses are known to be crucial in ALS symptoms [14]. Our study reveals that aberrant immune responses and the downregulation of metabolic activity are closely associated with neuronal death in neurodegenerative diseases. A variant analysis indicates that these processes are disrupted by genetic variants that interfere with normal splicing and gene expression, leading to the accumulation of misfolded proteins and increased inflammation. These disruptions exacerbate neuroinflammation and impair cellular energy metabolism, making neurons more susceptible to oxidative stress and apoptosis. The altered splicing affects the expression of key genes involved in immune regulation and metabolism, further contributing to neuronal damage. Thus, the interplay between immune dysregulation, metabolic dysfunction, and defective splicing represents a critical axis in the pathogenesis of ALS.

## 2. Methods

### 2.1. Dataset and Pre-Processing

We collected ALS samples (Supplementary Table S1) of two tissues comprising the frontal cortex (18 ALS and 9 control samples) and spinal cord (13 ALS and 8 control samples) [15,16] from the SRA database. The spinal cord samples are from sALS patients, while the frontal cortex samples include both sALS and c9ALS cases. These datasets include patients of age ranges from 36 to 84 and 55 to 82 for ALS and control samples, respectively. The outline of the work is represented as a flowchart in Supplementary Figure S1. RNA-seq reads were pre-processed using NGSQCToolkit [17], and reads were discarded when their PHRED score was less than 20. Adapter and hexamer contaminations were trimmed using FastqGroomer [18], Trim Galore [19], and FastqTrimmer [18]. Pre-processed reads were subjected to spliced read alignment using the hg38 human reference genome and the STAR [20] aligner. All methods were carried out in accordance with relevant guidelines and regulations.

### 2.2. Variant Analysis

Spliced read alignment yielded a higher rate of uniquely mapped reads, improving the accuracy of the variant analysis. We eliminated PCR duplicates using Rmdup [21] to avoid faulty read depth in variant calling. Variant calling was performed using samtools-Mpileup [22] and Varscan [23] with stringent criteria to detect variants that include (i) a Phred score > 30, (ii) minimum read depth of 10, (iii) transition/transversion ratio of more than 2, (iv) minimum allele frequency of 0.01, (v) p-adj < 0.001, (vi) strand bias evaluation (Fischer’s exact test) *p*-val < 0.01, (vii) mapping quality score > 30, and (viii) local alignment refinement. We categorized variants exclusively present in ALS samples but not in control subjects. The variants were annotated using ANNOVAR [24], which provides detailed information such as the gene name, genomic location, and known SNP information from dbSNP and COSMIC databases. We compared our variants with GWAS studies of other neurodegenerative diseases [25,26,27,28].

### 2.3. Differential Gene Expression Analysis

The transcriptome-based alignment was carried out using salmon [29]. Tximport [30] was used for quantifying transcript-to-gene abundance. The transcriptome-based alignments were considered for a differential gene expression analysis using DESeq2. We collected samples from the frontal cortex and spinal cord. C9ALS (a familial form associated with genetic mutations in the C9orf72 gene) and sALS (a sporadic form with no known genetic mutation) were included in our study. Differentially expressed genes (DEGs) were analyzed from samples obtained from both the frontal cortex and spinal cord for these two forms of ALS.

We accounted for technical variation and corrected the batch effect by dividing the batches into five batches (based on the region) and two conditions (control and disease). The five batches are (i) sALS (frontal cortex), (ii) c9ALS (frontal cortex), (iii) sALS (spinal cord), (iv) control samples (frontal cortex), and (v) control samples (spinal cord) with filtering criteria of a false discovery rate <0.05 and |log_2_foldchange| > 2.

Differential transcript expression refers to the variation in expression levels of different transcript isoforms of a gene. The composition and relative abundance of these isoforms are measured by Differential Transcript Usage (DTU). DTU identifies changes in the relative abundance of different transcript isoforms across conditions. It evaluates how the expression levels of various isoforms of a gene differ between experimental groups, allowing for insights into alternative splicing events and their functional implications by performing rigorous statistical tests in the tools (DRIMSeq and dTurte). Transcript level expression was analyzed using DRIMSeq [31] and dTurtle [32]. We filtered the transcripts that displayed differential expression patterns in both tools for a further downstream analysis.

### 2.4. Variant Effect Prediction

Identifying variants that affect regulatory processes contributing to complex disease traits is essential for understanding the disease mechanisms in ALS. The regulatory interplay of the variants has been predicted using various in silico tools. The effect of variants on miRNA binding, transcription factor binding, and epigenetic changes was analyzed. The information on miRNAs that were validated experimentally and their respective targets was obtained from miRDB [33] and miRTarbase [34]. The expression of miRNAs in ALS was retrieved from the literature [35]. The transcription factor-binding effect was predicted using SNP2TFBS [36]. The construction of regulatory networks was performed using the transcription factor expression in ALS from the literature and ReactomeFI [37]. QTLbase [38] was used to compare the variants associated with various quantitative trait loci.

### 2.5. Enrichment Analysis and Network Analysis of DEGs and VGs

It is essential to understand the functional relevance of genes and identify the pathways that participate in regulating biological functions. We used the following tools to perform pathway enrichment, EnrichR [39], clusterProfiler [40], and HumanBase [41], and CLUEGO [42] for visualization. Functional interaction networks and enrichment plots highlight the key genes involved in critical molecular functions, aiding in the understanding of disease mechanisms.

### 2.6. Metabolic Activity Prediction of Differentially Expressed Genes in Frontal Cortex and Spinal Cord Samples

METAflux [43] was used to predict the pathway scores based on normalized count data of ALS and control samples. METAFlux uses a human genome-scale metabolic model (GEM) that represents a standard model for understanding the relationship between genes, metabolites, and reactions. Expression profile data were used to predict the enzyme activity, infer nutrient levels, and calculate flux for the samples. This analysis was used to understand the metabolic activity of the differentially expressed genes and to analyze their effect on metabolic pathways involved in disease progression.

## 3. Results

### 3.1. Differential Gene Expression Analysis

**Figure 1:**
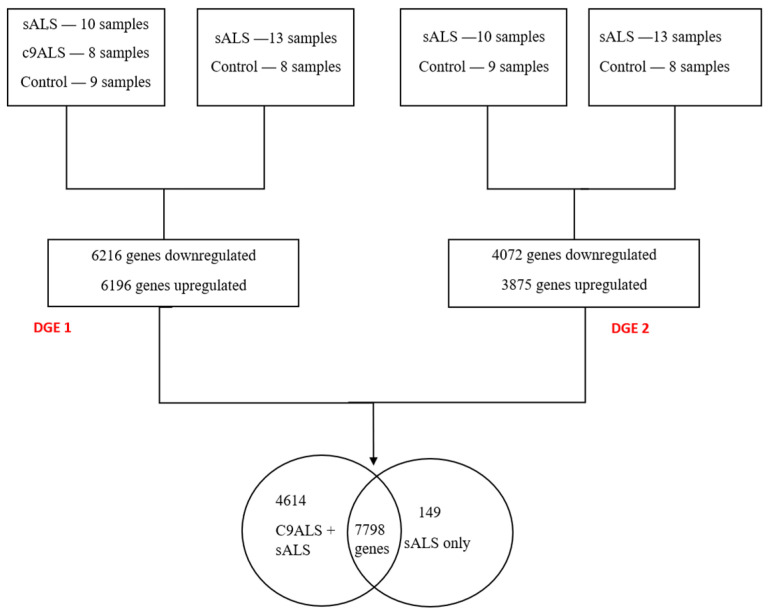
Differential gene expression analysis performed on frontal cortex and spinal cord samples.

**Figure 2:**
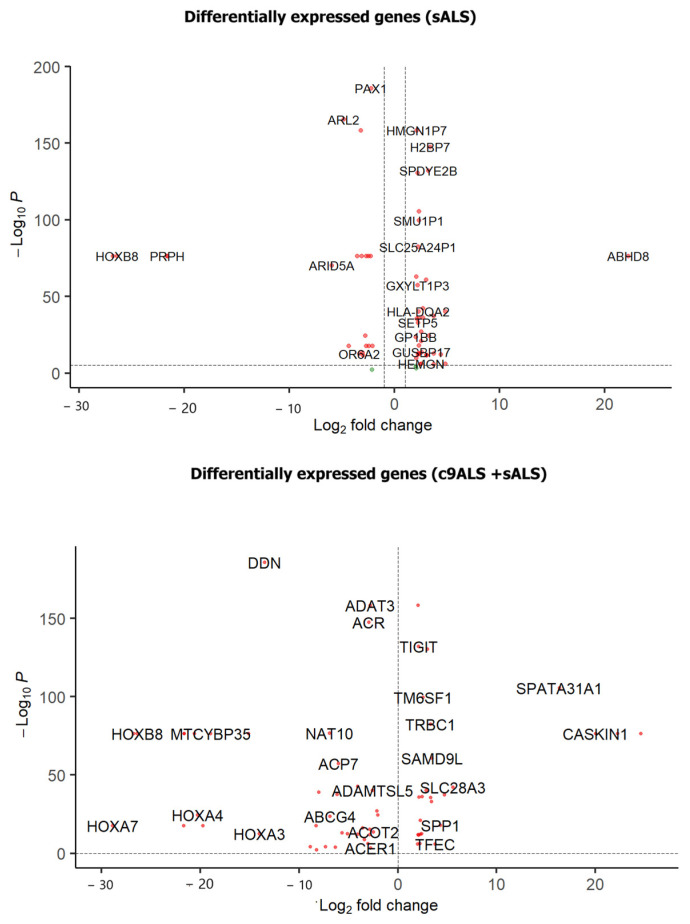
Volcano plots highlighting the DEGs identified in this study (the top plot represents genes in the DGE2 study and the bottom plot represents genes in the DGE1 study).

A differential gene expression analysis was conducted on samples from the frontal cortex (FC) and spinal cord (SC) using DESeq2. Two sets of analyses were performed (Figure 1): (i) involving both c9ALS and sALS samples and (ii) focusing solely on sALS samples from both tissues, hereafter referred to as DGE1 and DGE2 studies, respectively. This analysis identified 7798 differentially expressed genes across both sets, comprising 5597 novel genes and 2201 genes previously reported in the literature [44]. Figure 2 presents volcano plots that highlight key differentially expressed genes. Additionally, we mapped these differentially expressed genes (DEGs) to different cell types using CellMarker 2.0 [45]. We found that most of these DEGs are associated with excitatory neurons, reactive microglia, and astrocyte populations, indicating their significant involvement in non-neuronal cells.

### 3.2. Novel Gene Expression Patterns Identified Through the DEG Analysis

#### 3.2.1. Novel Genes of DGE1 (c9ALS+sALS) Study

Regarding the investigation into differential gene expression, encompassing both c9ALS and sALS samples derived from the frontal cortex, as well as sALS samples from the spinal cord, here, we discuss some important novel genes that may play pivotal roles in the pathophysiology of ALS, as outlined below.

***Histone methylation:*** The present study showed the upregulation of novel genes including DNMT3B (DNA methyltransferase 3B), EZHIP (EZH Inhibitory Protein), MACROH2A1 (MacroH2A.1 Histone), and MYB (myeloblastosis oncogene). These genes are reported as active regulators of histone methylation and chromatin remodeling processes, influencing downstream signaling genes. Furthermore, we identified variants (rs138663997, rs190560715) associated with the MACROH2A1 gene, which overlaps with the transcript, which consequently demonstrates changes in expression. Their elevated expression implies a potential role in modifying the epigenetic landscape, thereby influencing crucial cellular pathways linked to ALS pathology [46].

***Neuroinflammation:*** The NF-κB signaling pathway plays a critical role in neuroinflammation, being activated by various cellular stressors such as protein misfolding, metabolic demands, or injury. It serves as a pivotal factor in neurodegeneration, exhibiting a dual nature capable of both protecting and harming neuronal cells. Through our observations, we identified the upregulation of novel genes associated with immune response and inflammatory processes, many of which are key players in NF-κB signaling. These include CLEC4G (C-Type Lectin Domain Family 4 Member G), FOXP3 (forkhead box P3), IHH (Indian hedgehog), and NFKBID - Nuclear factor-kappa B Inhibitor delta (upregulated), as well as RFPL3 (Ret Finger Protein Like 3), PPM1N (Probable protein phosphatase 1N), and IRGM- Immunity Related GTPase M (downregulated), all of which regulate pro-inflammatory cytokines. The dysregulation of these genes in ALS suggests potential neuroinflammation, which could have adverse effects on neuronal function.

***Cerebral blood pressure regulation:*** Cerebral blood pressure regulation involves a delicate balance between vasoconstriction and vasodilation, crucial for maintaining optimal blood flow to the brain. We identified the dysregulation of genes associated with vasoconstriction, namely TACR2 (Tachykinin Receptor 2), TBXA2R (Thromboxane A2 Receptor), and PROK2(Prokineticin 2). These genes play key roles in smooth muscle cell contractions, directly influencing blood pressure regulation. The dysregulation of these genes disrupts the intricate mechanisms that govern vascular tone, leading to the abnormal constriction of blood vessels. This aberrant smooth muscle cell activity ultimately impacts cerebral blood flow dynamics and can contribute to fluctuations in blood pressure that affect neuronal activity and survival.

In summary, the analysis of differential gene expression patterns in the frontal cortex of ALS patients unveiled aberrant alterations in histone methylation and epigenetic regulation, alongside increased neuroinflammation and cerebral blood pressure dysregulation. These factors collectively exert significant effects on neuronal function, underscoring their importance in the pathogenesis of ALS.

#### 3.2.2. Novel Expression Signature of Common Genes from DGE1 and DGE2

We identified common differentially expressed genes between the frontal cortex and spinal cord ALS samples. The DEGs are majorly involved in metabolic processes and cell survival signaling pathways like NF-κB, WNT (Wingless-related integration site)/Notch signaling, and endocytosis. Here, we discuss some important novel genes from the above functions.

***NF-κB signaling:*** NF-κB signaling is implicated in the inflammatory response triggered by cellular stress factors, leading to the activation of reactive astrocytes and microglia, as well as the production of cytokines. In our investigation, we observed a dysregulation (upregulation) of genes involved in this pathway, namely BMP7 (bone morphogenetic protein 7), CCN3 (Cellular communication network factor 3), and CPNE1(Copine 1). It is supported with the fact that neuronal NF-κB signaling holds therapeutic potential for ALS [47].

***WNT/Notch signaling:*** This signaling pathway determines cell fate based on various factors and plays a crucial role in cell survival and death. Our study revealed the dysregulation of key genes involved in this pathway, including CSNK1E (Casein Kinase 1 Epsilon), WNT2 (WNT family member 2), DLK1 (Delta-like homologue 1), DLK2 (Delta-like homologue 2), and NOTCH2NLC((Notch 2 N-Terminal Like C), in both spinal cord and frontal cortex tissues. These findings highlight the significance of WNT/Notch signaling in ALS pathology across different regions of the central nervous system [48].

***Endocytosis:*** Endocytosis is vital for clearing cellular debris, disposing of misfolded proteins, supplying neurotrophic factors, and recycling neurotransmitters at the synapse. In our study, we observed the dysregulation of key genes involved in this process, including CCL17 (C-C Motif Chemokine Ligand 17), OLFM4 (Olfactomedin 4), and TFR2 (T ransferrin receptor 2). These findings underscore the importance of endocytosis in maintaining cellular homeostasis and synaptic function, implicating its potential role in the pathogenesis of ALS.

***Neuromuscular junction dysfunction:*** Neuromuscular junction (NMJ) dysfunction is a hallmark feature of ALS, characterized by impaired communication between motor neurons and muscle fibers. This dysfunction arises from genetic mutations, such as those affecting the SOD1 gene, which contribute to motor neuron degeneration, disrupt neurotransmitter release and transport, and trigger chronic inflammation and progressive muscle atrophy. In our study, we identified 22 genes (20 variant-associated genes) related to cell death and 13 genes (3 variant-associated genes) associated with a neuromuscular function that were upregulated, shedding light on the molecular mechanisms underlying NMJ dysfunction in ALS and highlighting potential targets for therapeutic intervention.

#### 3.2.3. Novel Genes of DGE2 (sALS) Study

Our analysis focuses on genes exclusively present in sporadic ALS (sALS) samples from the differential gene expression study.

***Protein degradation:*** We observed notable findings concerning the KBTBD13 (Kelch Repeat And BTB Domain Containing 13) gene. KBTBD13 is known to participate in the proteasome-mediated ubiquitination process, acting as an E3 ubiquitin ligase responsible for transferring ubiquitin molecules to target proteins, marking them for degradation. Our data indicated that KBTBD13 is upregulated in sALS samples, suggesting an increased activation of the protein degradation machinery. This upregulation may reflect increased levels of protein misfolding, prompting the activation of the ubiquitin–proteasome system as a cellular response to protein aggregation and dysfunction, characteristic features of ALS pathology [49].

***Actin filament dysfunction:*** Actin filaments play a crucial role in maintaining the structural integrity of neurons, preserving their shape, facilitating axonal transport of nutrients, and supporting synaptic functions. Alterations in actin filament dynamics can significantly impact neuronal functionality. In our study, we identified the ODAM (odontogenic ameloblast-associated) gene, associated with these essential functions, as dysregulated in ALS. This finding suggests a potential link between aberrant actin dynamics and the pathogenesis of ALS, highlighting the importance of understanding the molecular mechanisms underlying neuronal cytoskeletal dynamics in neurodegenerative diseases. A volcano plot of the genes highlighted here is represented in Supplementary Figure S2.

### 3.3. Differential Gene Expression Pattern Across Studies

We validated our differentially expressed genes by comparing the TPM (count) values of samples with GTEx-Brain tissue (TPM) data. There was a similar expression pattern (Supplementary Figure S3) observed among the GTEx (tissue) data and control samples of FC and SC. The expression of ALS samples varies from that of the controls and GTEx samples (other brain tissues). The comparison of differentially expressed genes among the frontal cortex and spinal cord samples was performed with reported single-cell RNA seq DEGs [50]. This comparison is performed to understand if the expression pattern synchronized with other studies reported earlier on ALS. The variation in expression signatures is shown in Supplementary Figure S3. The differentially expressed genes from single-cell studies consisted of various cell types including endothelial cells, oligodendrocytes, astrocytes, microglia, and VLMCs (vascular and leptomeningeal cells).

### 3.4. Differential Transcript Usage/Expression Analysis

Understanding the expression at the transcript level facilitates the classification of isoforms/transcripts contributing to phenotypic consequences in ALS disease. Transcript usage typically assists in identifying the differential composition, i.e., the proportion of transcripts between two conditions (ALS and control). Differential transcript expression indicates changes in the expression of at least one transcript of a gene between control and disease conditions. We examined the transcript usage and expression of differentially expressed genes in frontal cortex and spinal cord samples. We identified 41 significantly differentially expressed transcripts in both the frontal cortex and spinal cord samples. Subsequently, their proportions and expressions were investigated using the dTurtle package. The functional relevance of these transcripts was interpreted through enrichment studies, revealing that genes with significant transcript expression are involved in key processes such as immune response, epigenetic modifications, and stress response. Additionally, we identified variants overlapping with these transcript positions, which may potentially affect alternative splicing events and transcript proportions (obtained using Ensembl). The details of these variants are given in Supplementary Table S2. Here, we discuss some important genes with variant overlaps from the above functions.

***Immune response:*** BAX (BCL2-associated X, apoptosis regulator) is an apoptosis regulator that is upregulated in ALS and its activity has been reported previously [51]. In this study, one of its transcripts showed a significant differential expression. Additionally, BAX regulates immune response [52], which can induce inflammation associated with apoptotic activity. Transcript-specific changes are observed in XBP1 (X box binding protein 1), a positive regulator of immune and stress responses. This gene is already well known for its transcription factor activity in spinal cord samples [53]. The dysregulation of these pathways is implicated in neuroinflammatory conditions and affects immune homeostasis in the brain.

***Epigenetic modifications:*** The CDC73 (cell division cycle 73) gene is differentially expressed at the transcript level. One out of three transcripts are differentially expressed in this gene, which influences epigenetic regulation through its involvement in histone ubiquitination. It potentially impacts gene expression, which is crucial for neuronal function and survival [54,55]. Meanwhile, MIER2’s (MIER family member 2) differential expression and association with histone modifications suggest its role in modulating chromatin structure and gene expression patterns relevant to ALS pathogenesis [56]. Understanding how these genes contribute to epigenetic dysregulation in ALS may offer insights into disease mechanisms and potential therapeutic targets.

**Figure 3:**
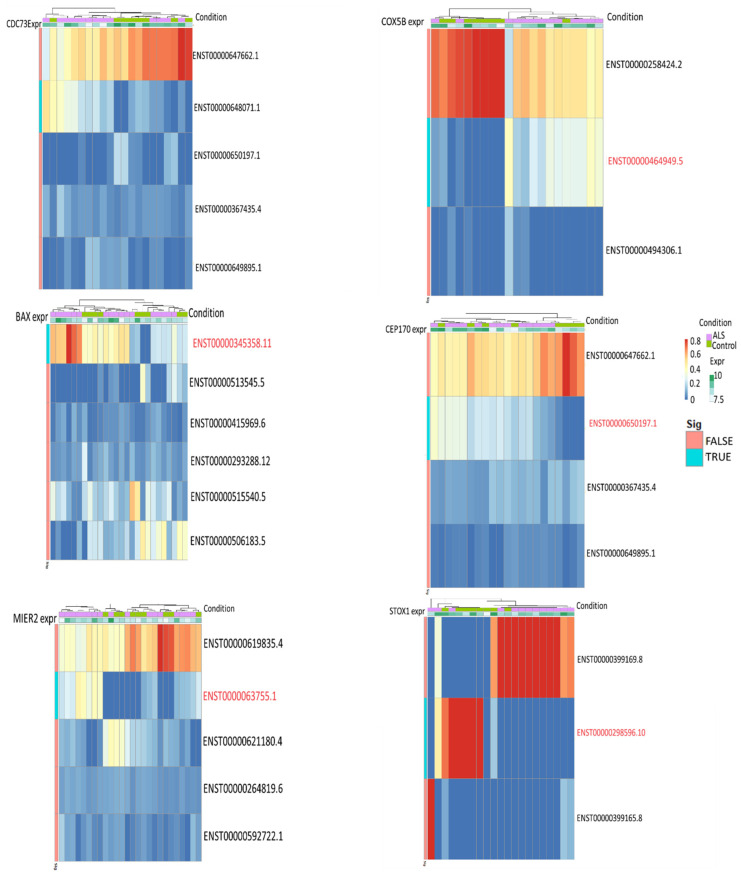
Transcript proportion plots of differentially expressed genes. The significant transcript in the genes is highlighted in red.

***Stress response:*** The differentially expressed genes in the spinal cord that are primarily associated with metabolic pathways are crucial for managing stress responses and metabolism. Notably, COX5B (cytochrome c oxidase subunit 5B) and CEP170 (centrosomal protein 170) exhibit significant changes in the transcript expression. COX5B plays a key role in cellular stress responses, while CEP170 is linked to autophagosome maturation, with its involvement in ALS highlighted in prior studies [57]. Additionally, STOX1 (storkhead box 1) displays significant differences in its transcripts and is known to participate in various cellular stress activities and mitochondrial functions [58]. In summary, the observed transcript changes in COX5B, CEP170, and STOX1 likely affect the stress response mechanisms in the spinal cord, potentially influencing cellular resilience and contributing to ALS pathogenesis. Transcript proportion plots with significant transcripts for the above genes are shown in Figure 3.

### 3.5. Variant Analysis of Frontal Cortex and Spinal Cord ALS Samples

In our study, we identified potential variants exclusive to disease samples and annotated them to understand their genomic distribution and overlapping transcripts. Variant calling was conducted on frontal cortex samples from both c9ALS and sALS patients to explore variant roles in ALS. Intronic variants predominated in c9ALS (35%) and sALS (28%) samples, as well as in spinal cord samples (35%). We further analyzed their involvement in regulatory effects and post-translational modifications using in silico tools, validated by the literature. Comparing 327 common variants between spinal cord and frontal cortex samples, we found that 44% of variant-associated genes were differentially expressed. Additionally, a comparison with disease-associated variants from DisGeNet and GWAS studies provided insights into their disease association. We predicted their contribution to gene regulatory effects using in silico tools, outlined in Supplementary Figure S4.

### 3.6. Variant Effect on Transcription Factor Binding

Variants potentially modify the binding of transcription factors, thereby influencing gene expression. In our study, variants in the frontal cortex and spinal cord were predicted to affect the binding of 190 transcription factors, with 33 of them showing differential expression. Through the enrichment analysis, we investigated the biological functions of these transcription factors, revealing their enrichment in majorly regulating stress responses and mediating immune response. Further details on specific transcription factors and their expressions are summarized in Supplementary Table S3, with their roles discussed here.

FOXO3 (forkhead box O3), FOXP1 (forkhead box P1), and NFE2L2 (nuclear factor, erythroid 2-like 2) are upregulated transcription factors that are known for their involvement in the regulation of response to ROS. This activity is crucial for neurodegeneration in ALS to handle oxidative stress and aid in finding therapeutic targets [59]. These genes are particularly enriched in the regulation of hydrogen peroxide-induced cell death, a significant contributor to motor neuron degeneration in ALS. BCL6 (B-cell lymphoma 6), FOXP1, and JUNB (JunB Proto-Oncogene) are key regulators of the immune response in the brain, each with distinct roles in modulating gene expression and immune cell function. BCL6 acts as a transcriptional repressor, while FOXP1 functions as a transcription factor involved in immune cell development. JUNB, a component of the AP-1 complex, regulates gene expression in response to immune signals. The dysregulation of these factors may disrupt immune homeostasis and contribute to neuroinflammatory conditions in ALS. In summary, the analysis reveals that many of the transcription factors affected by the variants are linked to functions related to immune and stress responses. Further investigation under cell type-specific conditions is warranted to gain deeper insights. Additionally, our study elaborates on the impact of these variants on other post-transcriptional regulatory mechanisms.

### 3.7. Variant Effect on miRNA Binding

miRNAs play a pivotal role in regulating gene expression by either promoting or inhibiting translation. Variants within miRNAs can disrupt miRNA binding, expression, and maturation processes, consequently affecting the expression of target genes. In our study, we illustrated how variant genes influence miRNA target genes, highlighting their enriched functionality in processes such as the DNA repair process, synaptic plasticity, and oxidative stress regulation. Table 1 represents the variants’ impact on miRNA binding and their respective target genes, with a detailed discussion on the role of these targets here.

Variant rs1844035 affects the binding affinity of miR-4477b, indirectly influencing the expression of its target genes. In ALS, downregulated miR-548aa’s binding is disrupted by variant rs771797645, impacting genes such as CITED (Cbp/P300 Interacting Transactivator With Glu/Asp Rich Carboxy-Terminal Domain) and SD2 (Skeletal Dysplasia 2) involved in the negative regulation of locomotion and response to oxidative stress [60,61]. This dysregulation highlights the symptomatic expression of these processes. Variant rs745666, associated with ALS, disrupts the binding of miR-548aa, which is downregulated in the disease. In ALS, miR-3615 targets two upregulated genes, KHSRP (KH-type splicing regulatory protein) and HIST1H1B (Histone cluster 1 H1 family member b), involved in neurotransmitter regulation and histone modifications, respectively [62,63]. Additionally, variant rs5432522 alters the binding of miR-548d-5p, downregulated in ALS, affecting its regulation of target genes EXT1 (exostosin glycosyltransferase 1) and SP1, enriched in processes such as cellular polysaccharide metabolism and angiogenesis [64,65]. Variant rs2427556 alters the binding of downregulated miR-941. Its target genes, DDB1 (damage-specific DNA binding protein 1) and NQO2 (N-ribosyldihydronicotinamide: quinone reductase 2), are involved in histone ubiquitination, metabolite regulation, and energy-related metabolic processes [66,67,68]. Previous studies provide evidence of the importance of these pathways in ALS [69,70].

Variant rs17091403 alters the binding of upregulated miRNA miR-2110 in ALS. MiR-2110 targets UNC5B (Unc-5 Netrin Receptor B), a downregulated gene in ALS that is responsible for negatively regulating the neuronal apoptotic process [71]. A reduced expression of UNC5B may lead to increased neuronal cell death activity. SETX (senataxin) and NEBL (nebulette) are targeted by miR-4477b, impacting the DNA repair process and synaptic plasticity, respectively [72,73]. The downregulation of these genes in ALS may compromise their functions, exacerbating disease pathology. Overall, identifying and studying the target genes of downregulated miRNAs is essential for validating disease-associated miRNA markers and understanding their role in ALS progression.

### 3.8. Variant Effect on Epigenetic Regulation

Epigenetic dysregulation plays a significant role in neurodegeneration, offering potential therapeutic targets through associated variants/genes. Both frontal cortex and spinal cord variants exhibit common epigenetic modifications, including expression regulation (eQTL), methylation, splicing, alternative polyadenylation (apaQTL), and RNA-editing quantitative trait loci. Among these, alternative polyadenylation influences gene expression by generating diverse 3′ ends, while RNA editing is influenced by variants (edQTLs), affecting disease risk/causing variants’ functional effects. Prioritizing the study of these variants and their roles in disease-associated pathways offers potential therapeutic markers, particularly since many variants in frontal cortex and spinal cord samples predominantly affect expression and splicing patterns. Increased eQTL variants indicate alterations in gene expression levels, potentially disrupting biological processes and contributing to disease pathogenesis. Similarly, a higher number of sQTL variants suggests changes in RNA splicing, which could lead to the production of aberrant protein isoforms with altered functions. Variant-associated genes (shown in Supplementary Table S4 [74,75,76,77,78,79,80,81]) that are involved in crucial functions like apoptosis, the regulation of metabolic processes, and neuroinflammation are discussed here.

In ALS, VSIG4 (V-set and immunoglobulin domain-containing 4) is downregulated due to the rs1044165 variant, impacting immune responses and suggesting a role in neuroinflammation [82]. The novel variant rs2259926 is associated with gene expression regulation, affecting ABHD12 (α/β hydrolase domain-containing protein 12), essential for lipid metabolism crucial for brain function, making it a potential therapeutic target for ALS. Additionally, SCAP (SREBF chaperone), linked to the eQTL variant rs10433549, regulates cholesterol homeostasis via SREBP1 signaling, potentially influencing neuronal health [83]. The dysregulation of these genes likely contributes to neurodegeneration in ALS.

Variant rs3758330 is associated with dysregulated splicing, impacting its associated gene ST6GALNAC6 (ST6 N-acetylgalactosaminide α-2,6-sialyltransferase 6), which plays a crucial role in glycoprotein metabolic processes essential for proper neuronal function. Dysfunctions in glucose transport and brain metabolism stress mitochondria, contributing to ALS neurodegeneration. Additionally, CDK11A (cyclin-dependent kinase 11A), downregulated in ALS and associated with variant rs10433549, regulates cell growth and mRNA metabolic processes crucial for neuronal proliferation, differentiation, and synaptic plasticity. The dysregulation of CDK11A expression may disrupt these processes, contributing to neuronal dysfunction and ALS progression. Information on these QTL variants and their expression is detailed in Supplementary Table S3. Overall, variant-associated genes play unfavorable roles in ALS progression by influencing splicing, expression regulation, methylation, RNA editing, and polyadenylation. DNM1L (dynamin 1-like), associated with the variant rs7966735 (apaQTL), is known for its involvement in mitochondrial apoptotic activity, crucial for maintaining mitochondrial health. Alternative cleavage and polyadenylation, linked to mitochondrial function, play a role in ALS pathogenesis. Additionally, TRAPPC9, associated with the variant rs199232, regulates forebrain development and synaptic vesicle trafficking, highlighting its importance in brain function and development, thus serving as a significant marker for investigating polyadenylation activity in ALS patients.

### 3.9. Function Enrichment on DEGs and Variant Genes

#### 3.9.1. Function Enrichment Network of Spinal Cord and Frontal Cortex DEGs

**Figure 4:**
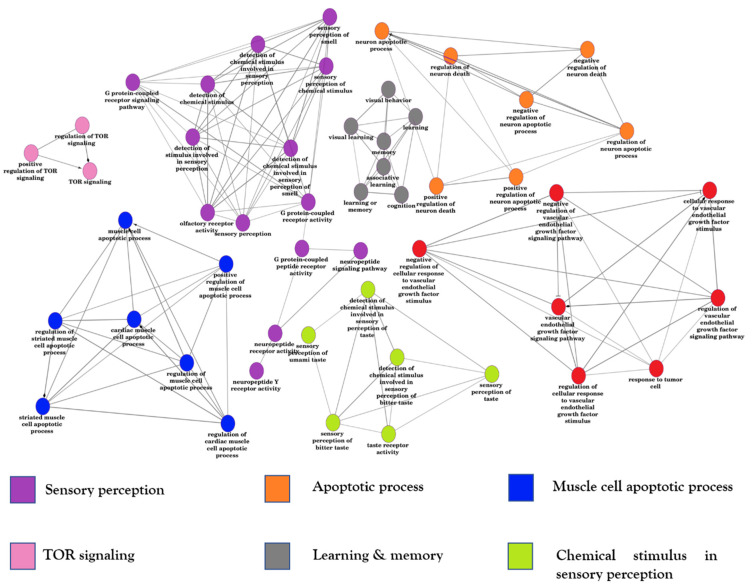
Function interaction network between common DEGs of DGE1 and DGE2.

We constructed a functional interaction network (Figure 4) using differentially expressed genes from ALS samples in the frontal cortex and spinal cord. The DEGs and the function/pathways are detailed in Supplementary Table S5 [84,85,86,87,88,89,90,91,92,93,94,95,96]. In this section, we highlight key processes’ genes that participate in sensory perception, muscle cell apoptosis, and TOR signaling.

***Sensory perception:***

Novel upregulated genes identified in our study, such as SPNS2 (SPNS Lysolipid Transporter 2), GNG13 (G protein subunit gamma 13), PDE4A (phosphodiesterase 4A), FOXG1 (Forkhead box G1), PNOC (Prepronociceptin), and OPN4 (Opsin 4), are actively involved in diverse sensory perception pathways. Considering the well-documented impact of sensory alterations in ALS patients [97], these genes provide valuable insights into their potential contributions to ALS symptoms. Furthermore, their association with variants helps unravel shared mechanisms underlying both sensory and motor symptoms in ALS. Recent research is focusing on deciphering patterns from these shared mechanisms [98]. These genes participate in various sensory perception pathways, including vision, olfaction, and pain sensation, by transmitting environmental sensory signals to the central nervous system for processing and interpretation. Additionally, some of these genes may regulate neurotransmitter release or signal transduction processes associated with sensory perception.

***Muscle cell apoptosis:***

POU6F1 (POU Class 6 Homeobox 1), TAFA5 (TAFA Chemokine Like Family Member 5), NPY5R (neuropeptide Y receptor type 5), ACTA1 (Actin Alpha 1, Skeletal Muscle), DBH (Dopamine Beta-Hydroxylase), and GHSR (growth hormone secretagogue receptor) genes share common functions related to muscle biology and neuromuscular regulation. They are involved in processes such as muscle cell differentiation, development, contraction, and apoptotic regulation. Upregulated in ALS, these genes play crucial roles in regulating muscle cell functions and are particularly associated with muscle differentiation, development, contraction, and apoptosis. The destruction of the neuromuscular junction, prominent in early ALS stages [99], is closely linked to striated muscle cell apoptosis, leading to the breakdown of muscles responsible for motor activities. Investigating these novel genes associated with ALS variants presents an opportunity to understand their role in controlling muscle degradation and its effects on neuromuscular junctions.

***TOR signaling:***

HTR6 (5-Hydroxytryptamine Receptor 6), LARS1 (Leucyl-TRNA Synthetase 1), LIN28A (Lin-28 Homolog A), PKHD1 (PKHD1 ciliary IPT domain containing fibrocystin/polyductin), RHEBL1 (Ras Homolog Enriched In Brain-Like Protein 1), RHEBP1 (RHEB Pseudogene 1), and ROS1 (ROS proto-oncogene 1) are genes that positively regulate TOR signaling, a vital pathway for muscle growth and maintenance. Therapeutic approaches targeting TOR signaling are being developed to address compromised neuromuscular junctions in ALS patients [100]. Ongoing experimental investigations seek to understand how TOR signaling influences protein aggregation and its associated genes. In summary, these genes, identified in both the spinal cord and frontal cortex, are functionally interconnected, potentially impacting disease progression in ALS.

#### 3.9.2. Gene Set Enrichment Analysis on DEGs

**Figure 5:**
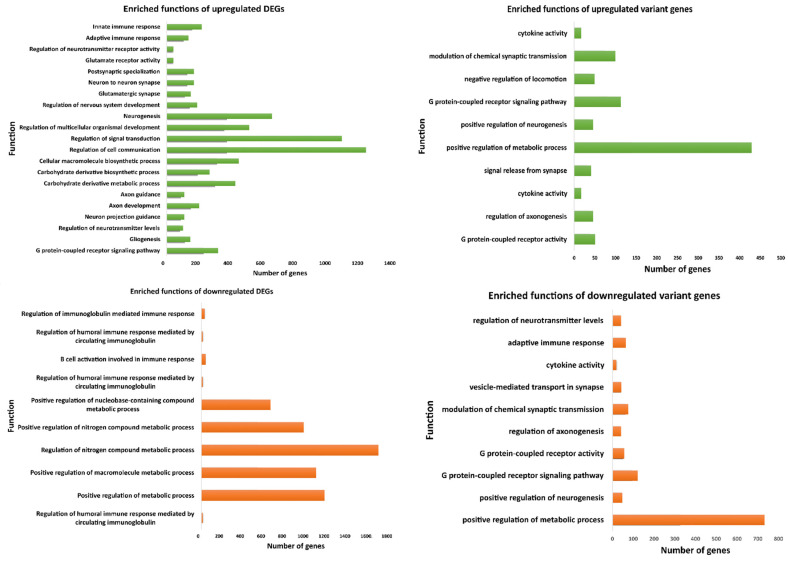
Enriched biological processes of differentially expressed genes and variant genes of our study.

To understand the functional impact of differentially expressed genes, we conducted an analysis of biological function enrichments. Our findings revealed that genes from both the frontal cortex and spinal cord are engaged in immune response activities and apoptotic functions (Figure 5). Specifically, upregulated genes are predominantly involved in immune response activities, while downregulated genes play roles in regulating metabolic processes.

***Immune response***

Histocompatibility genes are pivotal in orchestrating the immune response within the brain. They facilitate the recognition and presentation of antigens to T-cells, essential for initiating immune responses against pathogens or stressors. Furthermore, in the brain, histocompatibility genes regulate the activation and differentiation of immune cells, such as microglia and T-cells, in response to various stimuli, including infection, injury, or neurodegenerative processes. Additionally, histocompatibility genes modulate inflammation and immune surveillance within the central nervous system, impacting neuroprotection and neurotoxicity depending on the context.

The role of histocompatibility genes in the brain’s immune response is critical for maintaining homeostasis and defending against threats to neurological health. Moreover, genes like CD28 (Cluster of Differentiation 28), VSIG4 (V-set and immunoglobulin domain-containing 4), IL1A (interleukin-1 alpha), and IL1B (interleukin-1 beta) share common functions in regulating the immune response. CD28 facilitates T-cell activation and proliferation by providing a co-stimulatory signal. VSIG4, also known as V-set and immunoglobulin domain-containing protein 4, modulates T-cell activation and immune tolerance. IL1A and IL1B encode pro-inflammatory cytokines interleukin-1 α and β, respectively, mediating inflammatory responses and immune cell activation. Together, these genes play essential roles in coordinating the immune response, particularly in the regulation of inflammation and immune cell activity.

***Metabolic functions***

Metabolic genes like BECN1 are involved in the phospholipid metabolic process. It is observed that phospholipid metabolism has a role in motor neuron degeneration [101]. CBFA2T3 is an upregulated gene that is a major participant in regulating metabolic processes [102]. The further investigation of this gene can give more insights into using it for ALS disease therapeutics. Similarly, CBR1 (Carbonyl reductase 1), CBX7 (chromobox homolog 7), C1D (C1D Nuclear Receptor Corepressor), CARD11 (Caspase recruitment domain-containing protein 11), CARTPT (C ART Prepropeptide), and BMP8A (Bone morphogenetic protein 8A) are upregulated genes associated with our study’s variants that regulate most metabolic process activities. Gene therapy and knockout studies on metabolic genes can provide detailed information on their role in energy-deficient systems.

#### 3.9.3. Enrichment Study on Variant-Associated Genes

Variant-associated genes showed a similar pattern of differentially expressed genes by indulging in immune response and regulating metabolic activity. The variant genes were differentially expressed in our study and majorly regulated inflammation and interleukin mediating functions along with participation in pathways like GPCR signaling, Wnt signaling, cytokine-mediated signaling, and GPCR signaling pathways. The importance of immune response and inflammation has also been discussed previously [103,104,105,106,107]. These results show that inflammation and immune response have a crucial role in disease pathology.

Variant genes RPL3 (ribosomal protein L3), ZNF302 (zinc finger protein 302), and COLEC11 (collectin subfamily member 11) are upregulated in our study. These genes are observed to be involved in immune response, inflammation, cell adhesion, and neurotransmitter level regulation. The regulations of interleukin production, leukocyte-mediated immunity, and adaptive immune response are other immune-related functions in which the variant genes are active participants. There are studies denoting that MHCI expression in spinal cord motor neurons can save many neurons from degeneration [108]. The role of variant-associated genes in various regulatory effects has been discussed earlier. In summary, variant genes and the variants that act as different QTLs are capable of being crucial transcriptional and post-transcriptional regulators.

### 3.10. Metabolic Activity of DEGs

In this study, we combined gene expression data with a genome-scale metabolic network to predict metabolic flux distributions. This approach enables us to gain a system-level understanding of cellular metabolism. By analyzing flux distributions, we can identify metabolic pathways that are active or altered under disease conditions.

We assessed metabolite uptake and release scores based on the metabolic reactions involving the genes. Our analysis revealed a higher release of metabolites compared to uptake in disease samples, indicating potential metabolic dysregulation. By examining the uptake and release of compounds in metabolic reactions, we gain insights into nutrient utilization and cellular metabolic functions.

We used normalized counts from samples of the frontal cortex and spinal cord to predict pathway scores for various metabolic reactions. In the frontal cortex (Supplementary Figure S5), we observed an enrichment of β-oxidation in ALS samples, particularly in peroxisomes and mitochondria, which is associated with cellular stress and the formation of reactive oxygen species (ROS). Conversely, ROS detoxification appears to be more prominent in ALS samples in the frontal cortex, while it is enriched in control samples (Supplementary Figure S6) of the spinal cord. These findings suggest impaired metabolic activity in ALS samples, with identified pathways showing deviations in metabolic function. Additionally, fatty acid synthesis pathways are enriched in control samples. These are integral parts of brain function, including membrane structure, signaling, and neuroprotection. The detoxification of ROS free radicals is observed to be prominent in frontal cortex samples compared to spinal cord samples. The genes enriched in this pathway can have a neuroprotective effect on ALS patients and it is necessary to investigate their potential.

### 3.11. Limitations

A major limitation of the study is a lack of c9ALS samples from the spinal cord and a small size of the data obtained from both regions. Although a comparison of our expression pattern with GTEx and previous studies was performed, we still need a larger cohort and single-cell data to understand detailed expression at the gene/transcript level and corresponding variant associations.

## 4. Proposed Hypothesis and Discussion

We hypothesize that the aberrant immune response and decreased metabolic activity observed in neurodegenerative conditions are intricately linked to neuronal death. The dysregulation of immune pathways leads to chronic inflammation, which can exacerbate neuronal damage through the release of pro-inflammatory cytokines and the activation of microglia, further disrupting neuronal function. Concurrently, reduced metabolic activity impairs cellular energy production and disrupts vital metabolic pathways, leaving neurons vulnerable to oxidative stress and apoptosis. Together, these disturbances create a vicious cycle of inflammation and metabolic dysfunction, significantly accelerating neuronal degeneration. Targeting these intertwined pathways offers promising therapeutic opportunities to mitigate neurodegeneration and preserve neuronal health.

## 5. Conclusions

The principal insights of our study include the identification of novel differentially expressed genes (DEGs) in ALS, highlighting their crucial roles in regulating metabolic activity and neuroinflammation. Our study identified variant genes (CDC73, BAX, STOX1, MIER2) that overlap with significant differentially expressed transcripts, indicating their major contributions to the immune response and histone ubiquitination. The dysregulation of ubiquitination can lead to the accumulation of misfolded proteins, triggering inflammatory responses and contributing to neurodegeneration. This link underscores the interplay between the immune response and clearance defense mechanisms, emphasizing the importance of maintaining protein homeostasis. Additionally, we observed that upregulated genes primarily contribute to immune response and muscle contraction-related functions, while downregulated genes play crucial roles in regulating metabolic activity. The upregulation of immune response genes, combined with the downregulation of metabolic genes, exacerbates neuroinflammation and disrupts cellular energy metabolism, accelerating neuronal damage and death, thereby contributing to neurodegenerative disease progression. Targeting these dysregulated pathways may, therefore, offer potential therapeutic avenues to mitigate neurodegeneration.

We identified potential variants from transcriptome data obtained from the frontal cortex and spinal cord and compared them with QTL and GWAS studies. Most of the associated variants are involved in eQTL and sQTL studies, indicating that they potentially affect gene expression by altering splicing. Defective splicing can disrupt normal protein production and function, contributing to neuronal dysfunction and neurodegeneration. We identified common differentially expressed genes between the spinal cord and frontal cortex (7798, with 5597 upregulated genes and 2201 downregulated genes). These genes are primarily involved in NF-κB signaling, cell survival and death, and endocytosis pathways. This indicates that significant metabolic stress and immune response disrupt cellular homeostasis, affecting cell survival pathways. Genes involved in histone regulation and blood pressure regulation are specifically altered in the frontal cortex, while genes related to defective degradation processes are differentially expressed in the spinal cord. These findings suggest that distinct molecular pathways are disrupted in different regions, highlighting the complexity of neurodegenerative processes.

The novelty of our analysis included the transcription factor-binding modulation by variants and regulatory impacts of variants on the binding of miRNA and additional regulatory effects on the miRNA target genes. The detailed analysis unveiled the relationship between variants and differentially expressed transcripts (overlaps). Substantially, our investigation is a novel approach to previous datasets, as the variant-associated genes and the integration with transcripts were not previously studied.

In conclusion, the aberrant immune response and decreased metabolic activity observed in neurodegenerative conditions are intricately linked to neuronal death. The dysregulation of immune pathways leads to chronic inflammation [9], which can exacerbate neuronal damage through the release of pro-inflammatory cytokines and the activation of microglia, further disrupting neuronal function. Concurrently, reduced metabolic activity impairs cellular energy production and disrupts vital metabolic pathways, leaving neurons vulnerable to oxidative stress and apoptosis. Together, these disturbances create a vicious cycle of inflammation and metabolic dysfunction, significantly accelerating neuronal degeneration. Targeting these intertwined pathways offers promising therapeutic opportunities to mitigate neurodegeneration and preserve neuronal health.

## Figures and Tables

**Table 1:** Variant effect on miRNA binding and their targets.

Chr	Ref/Alt	SNP	Novel/Reported Variant	Affected miRNA (Expression)	miRNA Targets (Expression)
Chr10	C/T	rs17091403	Novel	miR-2110 (UP)	UNCB5 (DOWN)
Chr 9	G/A	rs1844035	Novel	miR-4477b (UP)	SETX, NEBL (DOWN)
Chr17	A/G	rs771797645	Novel	mir-548aa (DOWN)	CITED2, SOD2 (UP)
Chr17	G/C	rs745666	Novel	miR-3615 (DOWN)	KHSRP, HIST1H1B (UP)
Chr17	A/G	rs5432522	Novel	miR-548d-5p (DOWN)	EXT1, SP1 (UP)
Chr20	G/A	rs2427556	Novel	miR-941 (DOWN)	DDB1, NQO2 (UP)

## Data Availability

The datasets used in the study are available in the SRA database with the accession numbers SRP067645 (https://www.ncbi.nlm.nih.gov/sra/?term=SRP067645, accessed on 10 October 2024) and SRP056477 (https://www.ncbi.nlm.nih.gov/sra/?term=SRP056477, accessed on 10 October 2024).

## Supplementary Materials

The following supporting information can be downloaded at: https://www.mdpi.com/article/10.3390/genes15111431/s1, Supplementary Table S1: Sample information of ALS datasets; Supplementary Figure S1: Workflow of RNA-seq analysis using frontal cortex and spinal cord samples; Supplementary Figure S2: Volcano plot highlighting the novel genes from differential gene expression studies (DGE2); Supplementary Figure S3: Heatmap showing the variation in the gene expression pattern of ALS-associated genes; Supplementary Figure S4: Pipeline for filtering variants and grouping them based on their effect and distribution of QTL variants in frontal cortex and spinal cord samples; Supplementary Table S2: Variant genes with overlapped transcript (Differentially expressed transcript); Supplementary Table S3: Transcription factor information observed in ALS samples (FC and SC); Supplementary Table S4: QTL variants of frontal cortex and spinal cord samples; Supplementary Table S5: DEGs and their involvement in function/pathway; Supplementary Figure S5: METAFlux pathway scores of frontal cortex samples; Supplementary Figure S6: METAFlux pathway scores of spinal cord samples.
