# Supplementary material for: Tracing ALS Degeneration: Insights from Spinal Cord and Cortex Transcriptomes
Source: Genes (Basel). 2024 Nov 2;15(11):1431. doi: 10.3390/genes15111431 (PMC11593627; doi:10.3390/genes15111431)
Supplement: Supplementary file 1 [file genes-15-01431-s001.zip › genes-3281754-supplementary.pdf]

Supplementary Information

Tracing ALS degeneration: Insights from spinal cord and cortex transcriptomes

Nela Pragathi Sneha[1], S. Akila Parvathy Dharshini[1], Y-h. Taguchi[2] and

M. Michael Gromiha[1,*]

[1]Department of Biotechnology, Bhupat and Jyoti Mehta School of Biosciences,

Indian Institute of Technology Madras, Chennai 600036, Tamilnadu, India.

[2]Department of Physics, Chuo University, Kasuga, Bunkyo-ku, Tokyo 112-8551, Japan.

---

[*]corresponding author

Tel: +91-2257-4138

Fax: +91-2257-4102

E-mail: gromiha@iitm.ac.in

Supplementary Table S1: Sample information of ALS datasets

Study ID	Tissue	Number of ALS samples	Number of control samples
SRP067645	Spinal cord	sALS- 13	8
SRP056477	Frontal cortex	c9ALS -8; sALS -10	9

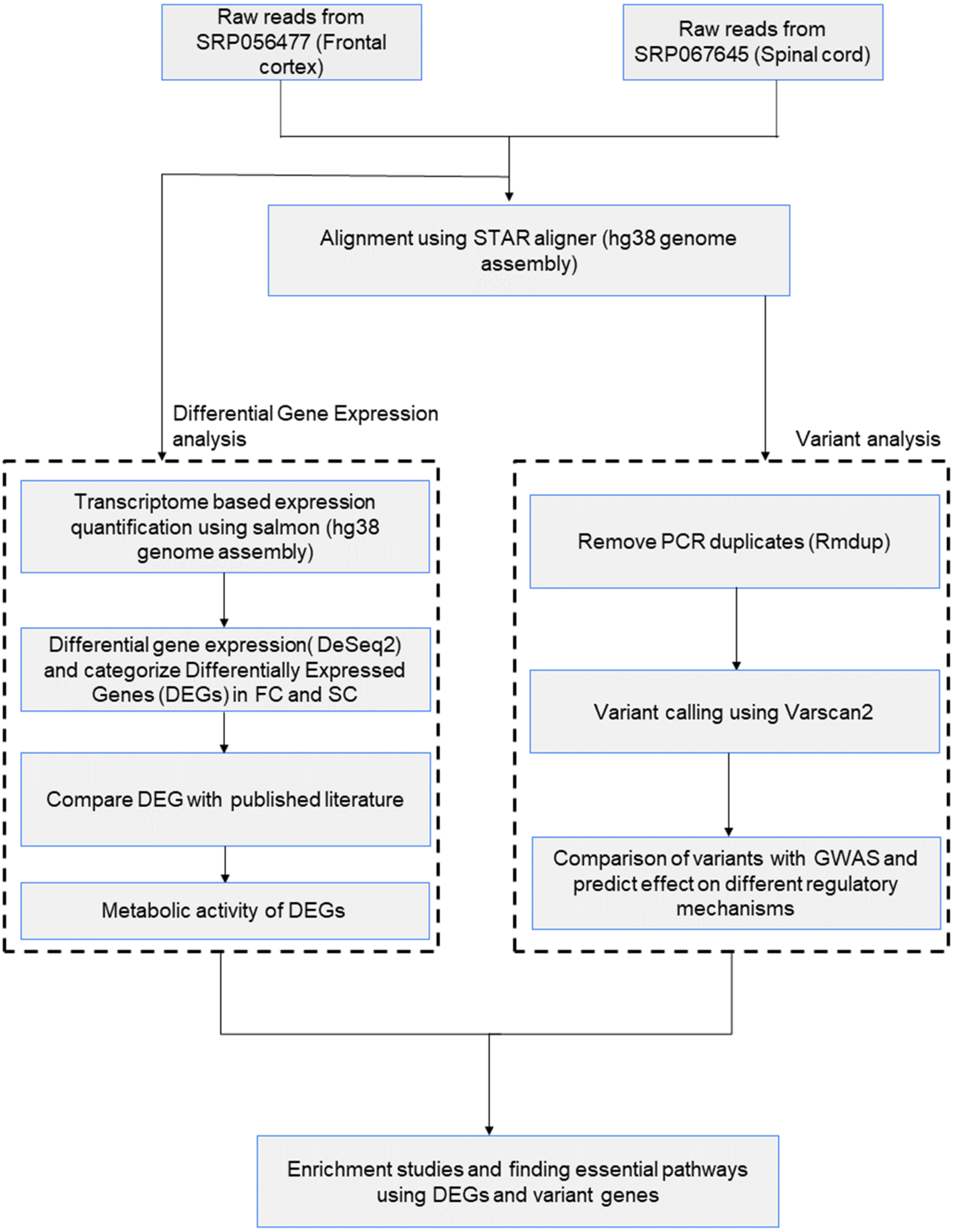

Supplementary Figure S1: Workflow of RNA-seq analysis using frontal cortex and spinal cord samples

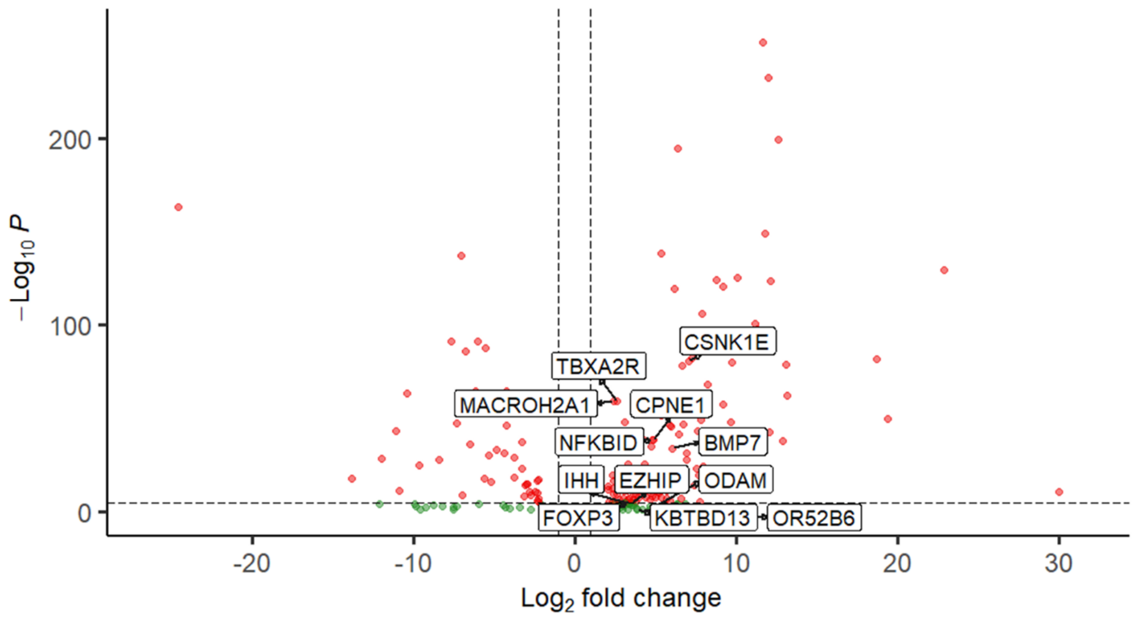

Supplementary Figure S2: Volcano plot highlighting the novel genes from differential gene expression studies (DGE2).

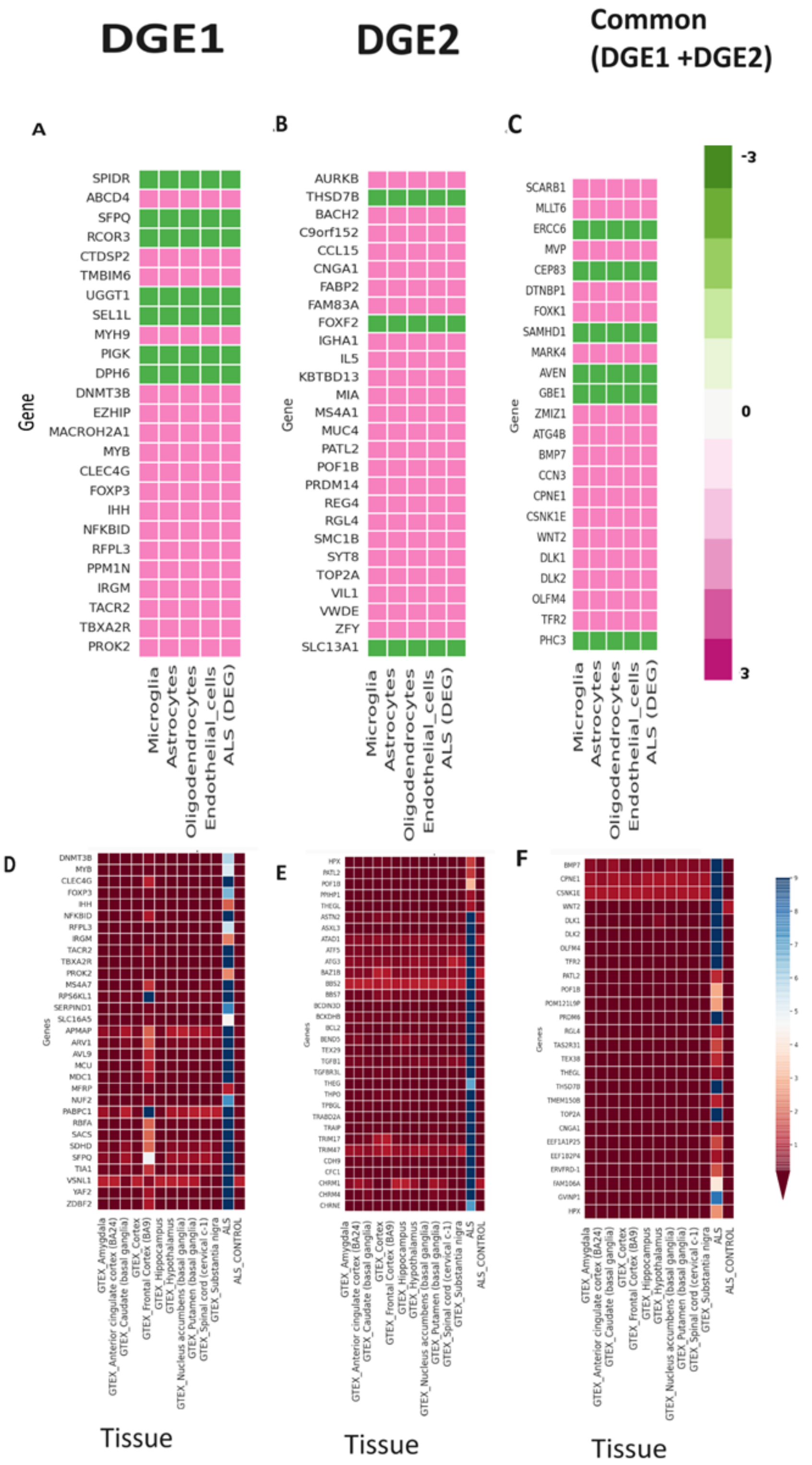

Supplementary Figure S3: Heatmap showing the variation in the gene expression pattern of ALS-associated genes across different studies and GTEx tissues (TPM) (A) DGE1 genes vs single cell genes, (B) DGE2 genes vs single cell genes, (C) Common genes of DGE1 and DGE2 vs single cell genes.

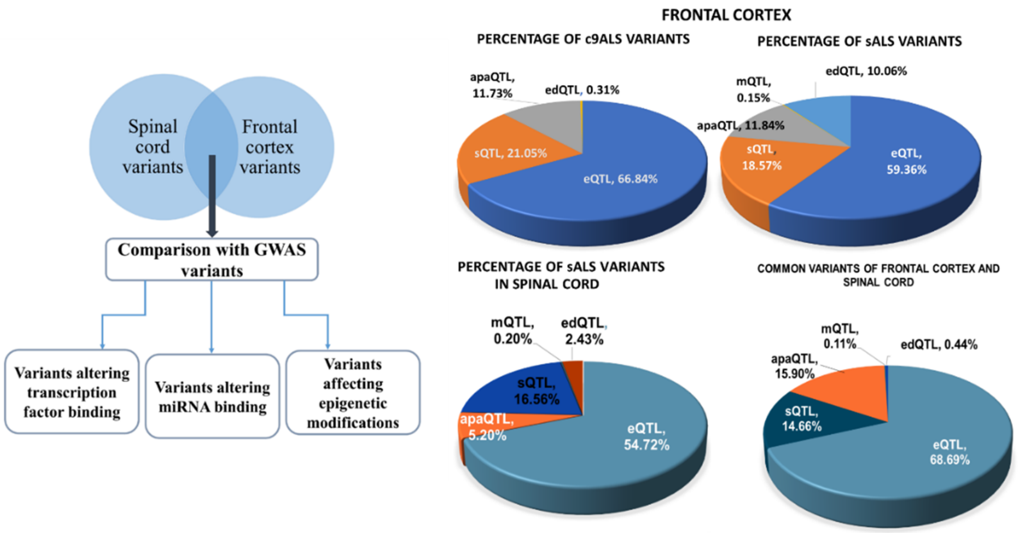

Supplementary Figure S4: Pipeline for filtering variants and grouping them based on their effect and distribution of QTL variants in frontal cortex and spinal cord samples

Supplementary Table S2: Variant genes with overlapped transcript (Differentially expressed transcript)

Chr	Ref/Alt	Variant	Gene	Overlapped transcript	Tissue	QTL study
1	T/A	rs7546869	CDC73(UP)	ENST00000648071.1	Frontal cortex	eQTL
1	T/C	rs28930673	CDC73(UP)	ENST00000648071.1	Frontal cortex	eQTL,pQTL
1	C/G	rs771545477	CDC73(UP)	ENST00000648071.1	Frontal cortex	NA
1	C/T	rs10921319	CDC73(UP)	ENST00000648071.1	Spinal cord	eQTL
19	G/A	rs55891703	MIER2(UP)	ENST00000635755.1	Frontal cortex	eQTL,mQTL
19	C/T	rs7256086	MIER2(UP)	ENST00000635755.1	Frontal cortex	eQTL,mQTL,pQTL
19	T/C	rs150312475	MIER2(UP)	ENST00000635755.1	Frontal cortex	eQTL,mQTL,sQTL
19	T/C	rs1009316	BAX (up)	ENST00000345358.11	Frontal cortex	eQTL,mQTL,sQTL
10	C/T	rs41278530	STOX1(UP)	ENST00000298596.10	Frontal Cortex	NA
10	C/T	rs10509306	STOX1(UP)	ENST00000298596.10	Frontal Cortex	eQTL,mQTL,sQTL,pQTL

Supplementary Table S3: Transcription factor information observed in ALS samples (FC and SC)

**Transcription factor**	**Expression**	**N_{fc}**	**N_{sc}**	**Function of TF**
FOXO3	UP	13	12	Regulation of response to ROS and hydrogen peroxide-induced cell death, neuron differentiation
FOXP1	UP	62	25	Regulation of response to ROS and hydrogen peroxide-induced cell death, alpha-beta T cell differentiation involved in immune response
NFE2L2	UP	21	6	Regulation of response to ROS and hydrogen peroxide-induced cell death
BLC6	UP	12	11	Alpha-beta T cell differentiation and activation are involved in immune response, neuron differentiation
JUNB	UP	14	7	Alpha-beta T cell differentiation and activation are involved in the immune response
RUNX1	UP	23	15	Regulation of CD4-positive, alpha-beta T cell differentiation and activation

Nfc: Number of variants altering its binding in frontal cortex; Nsc: Number of variants altering its binding in spinal cord.

Supplementary Table S4: QTL variants of frontal cortex and spinal cord samples.

**Chr**	**Ref/Alt**	**SNP**	**Variant**	**GE_{ALS}**	**QTL**	**Function**
Chr12	C/G	rs7966735	Novel	DNM1L (UP)	apaQTL	Apoptotic signaling pathway [74]
Chr8	T/C	rs199232	Novel	TRAPPC9(UP)	apaQTL	Forebrain/cortex development [75]
Chr20	T/C	rs2259926	Novel	ABHD12 (UP)	eQTL	Homeostasis [76]
Chr14	G/C	rs10141564	Novel	ALDH6A1(UP)	sQTL	Regulation of metabolic processes [77]
Chr9	T/C	rs3758330	Novel	ST6GALNAC6 (UP)	sQTL	Regulation of metabolic process [78]
ChrX	G/A	rs1044165	Novel	VSIG4 (DOWN)	eQTL	Inflammation (macrophage binding protein) [79]
Chr1	G/A	rs61073446	Novel	CDK11A (DOWN)	sQTL	Regulation of mRNA metabolic process [80]
Chr3	C/T	rs10433549	Novel	SCAP (DOWN)	eQTL	Regulation of sterol depletion [81]

Supplementary Table S5: DEGs and their involvement in function/pathway

Pathway/function	DEGs	DEG study(c9ALS+sALS; sALS only)	References for pathway/function in ALS
WNT/Notch signaling	CSNK1E, WNT2, DLK1, DLK2, NOTCH2NLC,	DGE1 and DGE2	Neuroprotective effect - Notch pathways [84]
TOR signalling	HTR6, LARS1, LIN28A, PKHD1, RHEBL1, RHEBP1, ROS1	DGE1	Cell growth and aging [85]
Histone methylation	DNMT3B, EZHIP, MACROH2A1, MYB	DGE1	Effects on regulating gene transcription [86]
Muscle cell apoptosis	POU6F1, TAFA5, NPY5R, ACTA1, DBH, GHSR	DGE1	Muscle atrophy in ALS model [87]
Neuroinflammation	CLEC4G, FOXP3, IHH, NFKBID	DGE1	Neurotoxic effect in ALS [88]
Neuromuscular junction dysfunction	SOD1, DNAJC5<br>EFNA2<br>FCHSD1<br>FCHSD2<br>HDAC4<br>ITGB1	DGE1	NMJ dysfunction in ALS [89]
Sensory perception	HTR6, LARS1, LIN28A, PKHD1, RHEBL1, RHEBP1, ROS1	DGE1	Sensory neurons in ALS [90]
Cerebral blood pressure regulation	TACR2, TBXA2R, PROK2	DGE1	Cerebral uptake and BBB permeability in ALS [91]
Immune response	BAX, XBP1	DGE1	Neuroprotective inflammation in ALS [92]
NF-κB signaling	BMP7, CCN3, CPNE1	DGE1	NF-κB in microglia - neuroinflammation [93]
Stress response	CEP170,<br>COX5B,<br>STOX1	DGE1	Stress response in ALS [94]
Protein degradation	KBTBD13	DGE2	Protein degradation in ALS [95]
Epigenetic modifications	MIER2,CDC73	DGE1	Epigenetic changes in ALS [96]

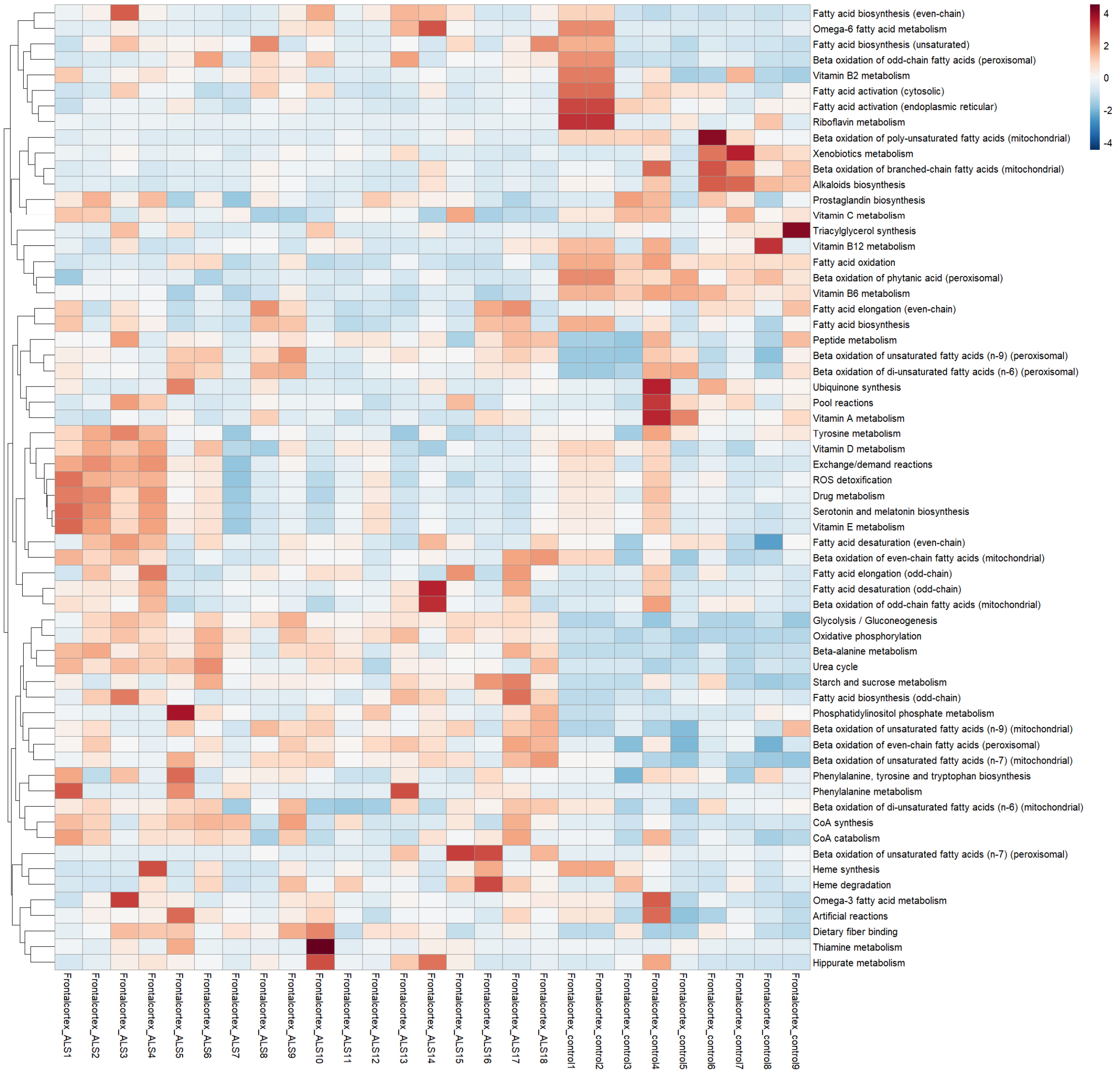

Supplementary Figure S5: METAFlux pathway scores of frontal cortex samples

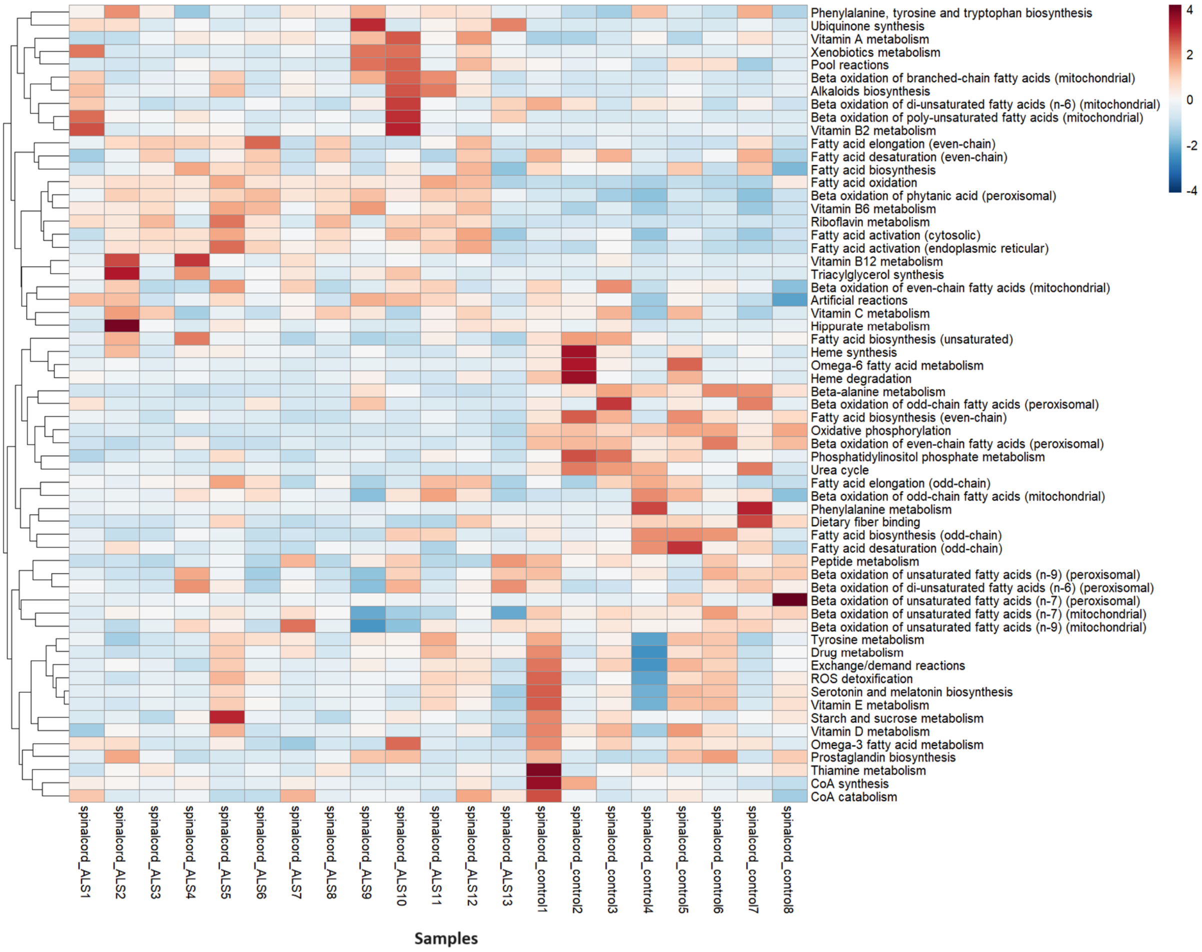

Supplementary Figure S6: METAFlux pathway scores of spinal cord samples
